# Twist1- and Twist2-Haploinsufficiency Results in Reduced Bone Formation

**DOI:** 10.1371/journal.pone.0099331

**Published:** 2014-06-27

**Authors:** Yanyu Huang, Tian Meng, Suzhen Wang, Hua Zhang, Gabriele Mues, Chunlin Qin, Jian Q. Feng, Rena N. D'Souza, Yongbo Lu

**Affiliations:** 1 Department of Biomedical Sciences and Center for Craniofacial Research and Diagnosis, Texas A&M University Baylor College of Dentistry, Dallas, Texas, United States of America; 2 The State Key Laboratory Breeding Base of Basic Science of Stomatology (Hubei-MOST) & Key Laboratory of Oral Biomedicine, Ministry of Education, School & Hospital of Stomatology, Wuhan University, Wuhan, China; 3 The University of Utah School of Dentistry, Salt Lake City, Utah, United States of America; Inserm U606 and University Paris Diderot, France

## Abstract

**Background:**

Twist1 and Twist2 are highly homologous bHLH transcription factors that exhibit extensive highly overlapping expression profiles during development. While both proteins have been shown to inhibit osteogenesis, only Twist1 haploinsufficiency is associated with the premature synostosis of cranial sutures in mice and humans. On the other hand, biallelic Twist2 deficiency causes only a focal facial dermal dysplasia syndrome or additional cachexia and perinatal lethality in certain mouse strains. It is unclear how these proteins cooperate to synergistically regulate bone formation.

**Methods:**

Twist1 floxed mice (*Twist1* ^f/f^) were bred with Twist2-Cre knock-in mice (*Twist2* ^Cre/+^) to generate Twist1 and Twist2 haploinsufficient mice (*Twist1* ^f/+^; *Twist2* ^Cre/+^). X-radiography, micro-CT scans, alcian blue/alizarin red staining, trap staining, BrdU labeling, immunohistochemistry, *in situ* hybridizations, real-time PCR and dual luciferase assay were employed to investigate the overall skeletal defects and the bone-associated molecular and cellular changes of *Twist1* ^f/+^;*Twist2* ^Cre/+^ mice.

**Results:**

Twist1 and Twist2 haploinsufficient mice did not present with premature ossification and craniosynostosis; instead they displayed reduced bone formation, impaired proliferation and differentiation of osteoprogenitors. These mice exhibited decreased expressions of *Fgf2* and *Fgfr1–4* in bone, resulting in a down-regulation of FGF signaling. Furthermore, *in vitro* studies indicated that both Twist1 and Twist2 stimulated 4.9 kb *Fgfr2* promoter activity in the presence of E12, a Twist binding partner.

**Conclusion:**

These data demonstrated that *Twist1*- and *Twist2*-haploinsufficiency caused reduced bone formation due to compromised FGF signaling.

## Introduction

Mammalian Twist1 and Twist2 are two members of the Twist subfamily of the basic-helix-loop-helix (bHLH) transcription factors that have been highly conserved during evolution [1]. In *Drosophila*, a single *Twist* gene, *DTwist*, is essential for embryonic gastrulation and mesodermal formation [2], [3]. Mouse Twist1 was identified by its high homology with *DTwist* [4], [5], while Twist2, originally called “Dermo1”, was discovered by a yeast-two-hybrid screen using the ubiquitous bHLH protein E12 as bait [6]. The expression patterns of Twist1 and Twist2 show an extensive overlap during mouse embryonic development [6], and their encoded proteins exhibit a high degree (up to 98%) of sequence similarity [7]. Both proteins perform various functions by forming either homodimers or heterodimers with bHLH E proteins (E12/E47) that bind to DNA canonical regulatory sequences called “E-boxes” (CANNTG) in the promoter region of target genes [8].

In humans, mutations in the *TWIST1* gene are associated with Saethre-Chotzen Syndrome (SCS), which is an autosomal dominant disorder characterized by craniosynostosis, brachydactyly, soft tissue syndactyly and facial dysmorphism [9]. The skeletal phenotype of Twist1-heterozygous mouse consistently resembles that of human SCS with premature fusion of the cranial suture [9], [10]. As mouse embryonic development progresses, the Twist1 expression declines in the developing bones of the skull [11]. In addition, Twist1 overexpression was found to inhibit osteoblast differentiation *in vitro* and *in vivo* [12], [13], [14]. Together, these observations suggest that Twist1 negatively regulates osteoblast differentiation and bone formation.

Various molecular mechanisms may be responsible for the inhibitory role of Twist1 in osteoblast differentiation. Twist1 may modulate FGF signaling, especially *Fgfr2* expression in cranial suture development [15], [16], [17] or it may directly bind to and inhibit the transactivation function of Runx2, a master regulator of osteogenesis [11]. In addition, Twist1 might indirectly regulate the *Runx2* expression through modulating *FGFR2* expression as shown in the *ex vivo* cultured primary osteoblasts isolated from human SCS patients [18]. Finally, it is possible that Twist1 inhibits osteoblast apoptosis via the suppression of TNF-α expression [19].

Twist2 has been shown to have an inhibitory function similar to that of Twist1 in bone formation [11]. While recessive *TWIST2* mutations in humans and its inactivation in mice result in a focal facial dermal dysplasia (FFDD) syndrome, there is no Twist2-deficient skeletal phenotype [20]. The phenotypic difference between the Twist1- and Twist2-deficient subjects is indeed intriguing when viewed in the context of their significantly overlapping expression patterns *in vivo* [6] and their similar functions in bone formation [11]. Thus, it is largely unknown how Twist1 and Twist2 synergistically regulate bone formation and what molecular mechanism is involved.

In this study, we generated a compound *Twist1*- and *Twist2*-haploinsufficient animal model, *Twist1^flox/+^*; *Twist2^Cre^* ^/+^ mice, by crossing Twist1 floxed mice with Twist2 *Cre* knock-in mice. Thus, the compound *Twist1^flox/+^*; *Twist2^Cre^* ^/+^ mice had one allele of *Twist2* replaced by the *cre* recombinase and one allele of *Twist1* deleted specifically in tissues where the *Twist2* gene was expressed. To our surprise, we found that the *Twist1^flox/+^*; *Twist2^Cre^* ^/+^ mice exhibited reduced bone formation and impaired proliferation and differentiation of osteoprogenitors. The skeletal abnormalities were associated with reduced FGF signaling as a consequence of the decreased expressions of *Fgf2* and *Fgfrs*.

## Materials and Methods

### Ethical Approval

All animal protocols were approved by the Institutional Animal Care and Use Committee (IACUC) at Texas A&M University Baylor College of Dentistry. IACUC has specifically given ethical approval for all the procedures in this study.

### Generation of Twist1^flox/+^; Twist2^Cre/+^

*Twist1* floxed mice (*Twist1^flox/flox^*) were maintained on a C57/129 mixed genetic background [21] and *Twist2 Cre* Knock-in mice (*Twist2^cre/+^*) were on a 129 genetic background [22]. The *Twist1^flox/flox^* mice were bred with *Twist2^cre/+^* mice to generate compound *Twist1^flox/+^*; *Twist2^cre/+^* mice. The *Twist2^Cre/+^* mice carry a Cre recombinase that replaces one allele of the *Twist2* gene [22]; therefore, the floxed *Twist1* allele is deleted in the tissues where the *Twist2* gene is active. The *Twist1* floxed mice and *Twist2^Cre/+^* mice were genotyped as described previously [21], [22]. In this study, we analyzed the skeletal phenotype of 6–8 day-old *Twist1^flox/+^*; *Twist2^Cre^* ^/+^ mice and used age-matched *Twist1^flox/+^*; *Twist2^+/+^* as control mice (counting the day of birth as day 0).

### Alcian blue/alizarin red staining of the skeleton

Alcian blue/alizarin red staining was performed to analyze the overall skeletal and mineralization defects, as described previously [23]. Briefly, 6-day-old *Twist1^flox/+^*; *Twist2^cre/+^* mice and control mice were sacrificed, skinned, eviscerated and fixed for three days in 95% ethanol. They were then stained with alcian blue for cartilage and alizarin red for bone visualization.

### Plain X-ray radiography and high-resolution microcomputed tomography (μ-CT)

The femurs and tibiae from 6-day-old *Twist1^flox/+^*; *Twist2^cre/+^* mice and control mice were dissected free of the skeletal muscles and fixed in 70% ethanol. For plain X-ray radiography, the femurs were analyzed with a Faxitron MX-20 specimen radiography system (Faxitron X-ray Corp., Buffalo Grove, IL) as described previously [24]. For the high-resolution μ-CT analyses, the tibiae were scanned at 3.5-µm resolution using a μ-CT35 imaging system (Scanco Medical, Basserdorf, Switzerland), as previously described [24]. The trabecular bone was analyzed at a threshold of 160 in 20 sections underneath the growth plate.

### Histology, immunohistochemistry and in situ hybridization

For histologic analysis, the bone specimens from 6-day-old mice were fixed in freshly prepared 4% paraformaldehyde, decalcified in 10% EDTA with 0.5% paraformaldehyde, and embedded in paraffin using standard procedures [25]. Serial 7-µm sections were cut and mounted on silane-coated slides. The sections were then used for Hematoxylin and Eosin (H&E) staining, Tatrate-resistant alkaline phosphatase (TRAP) staining, immunohistochemistry or *in situ* hybridization, as described previously [24], [25].

The following antibodies were used for the immunohistochemical analyses: anti-Osterix (Santa Cruz Biotechnology, Dallas, TX, USA; 1∶400), rabbit anti-biglycan antibody “LF-159” (a gift from Dr. Larry Fisher, National Institutes of Health, Bethesda, MD; 1∶1000), anti-Fgfr2 polyclonal antibody (Abcam, Cambridge, MA, USA; 1∶5000), and anti-phospho-p44/42 MAPK (Erk1/2) antibody (Cell Signaling Technology, Danvers, MA, USA; 1∶100). All the immunohistochemical experiments were performed with a 3, 3-diaminobenzidine kit (Vector Laboratories, Burlingame, CA) according to the manufacturer's instructions.

For the *in situ* hybridization, the RNA probes for dentin matrix protein (*Dmp1*), osteocalcin (*Ocn*) and alkaline phosphatase (*Alp*) were labeled with digoxigenin (DIG) using a RNA Labeling Kit (Roche, Indianapolis, IN, USA) according to the manufacturer's instructions. DIG-labeled RNA probes were detected by an enzyme-linked immunoassay with a specific anti-DIG-alkaline phosphatase antibody conjugate (Roche, Indianapolis, IN) and a VECTOR NBT/BCIP alkaline phosphatase substrate (Vector Laboratories, Burlingame, CA), which produced a blue color indicating positive signals. Methyl green was used for counterstaining.

### Cell proliferation assay

Six-day-old *Twist1^flox/+^*; *Twist2^cre/+^* mice and control mice were intraperitoneally injected with 5-bromo-2′-deoxyuridine (BrdU) (10 mg/100 g body weight) at 24 hours and then two hours before sacrifice. The long bones were collected and processed for paraffin sectioning as described above. The incorporated BrdU was detected with a BrdU staining kit (Invitrogen, Camarillo, CA, USA) according to the manufacturer's instructions. The BrdU-positive nuclei and total nuclei were counted in the metaphysis region (trabecular bone) as well as in the midshaft (cortical bone). The data represented the percentage of BrdU-positive nuclei from four individual animals each group.

### Quantitative Real-time PCR

Total RNA was extracted using Trizol (Invitrogen, Camarillo, CA, USA) from the long bones of 8-day-old Twist1/2 dHet mice and control mice and reverse-transcribed into cDNA with a Reverse Transcription Kit (QIAGEN, Germantown, MD, USA). Quantitative real-time PCR (qPCR) was performed using a Go Tag qPCR Master Mix System (Promega, Madison, WI, USA). Mouse 18S rRNA primers (PPM57735E-200, QIAGEN, Germantown, MD, USA) were used for normalization. The primers for *Alp*, *Ocn*, bone sialoprotein (*Bsp*), osterix (*Osx*), *Dmp1* and *Runx2* were reported elsewhere [26], [27]. The primers for *Fgf2*, *Fgfr1*, *Fgfr2*, *Fgfr3*, *Fgfr4*, *Erm* (*Etv5*) and *Pea3* (*Etv4*) are listed in Table 1. The primers for *Fgfr2* were specifically designed to amplify the mesenchymal isoform of *Fgfr2* (*Fgfr2-IIIc*). All experiments were performed in triplicate on three animals. The data were analyzed using the 2^−ΔΔCT^ method as described previously [28].

**Table 1:** Primers used for real-time PCR.

Gene	Forward primer (5′-3′)	Reverse primer (5′-3′)
*Fgf2*	agcggctctactgcaagaac	gccgtccatcttccttcata
*Fgfr1*	ttcagtggctgaagcacatc	gcagagtgatgggagagtcc
*Fgfr2*	gtgcttggcgggtaattcta	gatgactgtcaccaccatgc
*Fgfr3*	ccaccttcaagcagttggtag	gggtgaacaccgagtcatct
*Fgfr4*	aagtcatccgtggccactac	cgagaggcaggtctagattca
*Erm*	gatgatgcctgaaagccagt	gggaccccatgttcatacag
*Pea3*	cctatgactcccccagacaa	cctccctgaggagatgtgaa

### Cell culture, constructs and promoter luciferase assay

The C3H10T1/2 mesenchymal cells and MC3T3-E1 preosteoblast cells were cultured as described previously [29]. A 4.9 kb-*Fgfr2* promoter luciferase construct and expression constructs for Twist1 and E12 have been reported elsewhere [30]. An expression construct for Twist2 was generated by cloning Twist2 cDNA into the BamHI and EcoRI sites of pcDNA3 vector (Invitrogen). The promoter luciferase assay was performed as described previously [24], [30]. Briefly, C3H10T1/2 cells and MC3T3-E1 cells were plated in 24-well plates at a density of 3×10^4^ cells per well. Then the cells were transiently transfected with 0.1 µg of 4.9 kb-*Fgfr2* promoter luciferase construct, together with 0.4 µg of various constructs expressing Twist1, Twist2 or E12 using FuGENE 6 Transfection Reagent (Roche, Indianapolis, IN, USA). The total amounts of transfected DNA were balanced by the addition of an empty vector (pcDNA3). A *Renilla* luciferase expression construct was co-transfected as an internal control to monitor the transfection efficiency. Forty-eight hours later, the transfected cells were analyzed using a dual luciferase reporter assay system (Promega, Madison, WI, USA). The luciferase activities were normalized by the control. All experiments were carried out in triplicate and repeated three times.

### Statistical analysis

The statistical analyses were performed with a one-way ANOVA for a multiple group comparison and the Student's *t*-test for a two-group comparison. If significant differences were found with the one-way ANOVA, the Student's *t*-test was used to determine which groups were significantly different from the others. The quantified results were expressed as mean ± standard deviation (SD). P<0.05 was considered to be statistically significant.

## Results

### Generation of Twist1- and Twist2-haploinsufficient mice

*Twist1^flox/+^*; *Twist2^Cre/+^* mice were generated by breeding *Twist1* floxed mice (*Twist1^flox/flox^*) with *Twist2 Cre* Knock-in mice (*Twist2^cre/+^*), in which one allele of *Twist2* was replaced by the Cre recombinase. Twist2-Cre is active in the condensed mesenchyme that will later produce the chondrocytes and osteoblasts [22]; therefore, in the compound *Twist1^flox/+^*; *Twist2^Cre/+^* mice, the floxed *Twist1* allele is deleted in the osteoblasts and their precursors. Consequently, both Twist1 and Twist2 were haploinsufficient in the osteoblast lineage of the *Twist1^flox/+^*; *Twist2^Cre/+^* mice. Real-time PCR confirmed that the mRNA levels of both *Twist1* and *Twist2* were about three folds less in the *Twist1^flox/+^*; *Twist2^Cre/+^* mice than in the control mice (Figure S1). Although the floxed *Twist1* allele was conditionally deleted, the compound *Twist1^flox/+^*; *Twist2^Cre^* ^/+^ mice had a phenotype similar to that of the mice completely heterozygous for both the *Twist1* and *Twist2* genes [31]. Most of them died within two weeks after birth but a few of them survived to adulthood and were fertile.

### Reduced bone formation in Twist1^flox/+^; Twist2^Cre/+^ mice

**Figure 1:**
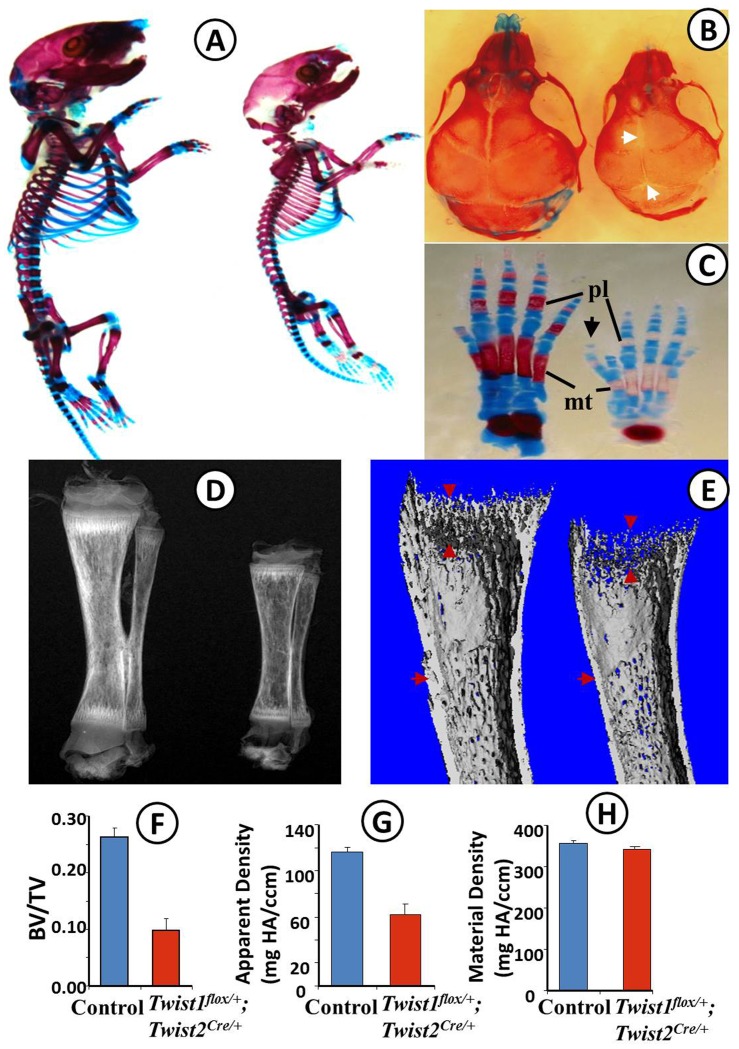
Reduced bone formation in *Twist1^flox/+^*; *Twist2^Cre/+^* mice. (A) Skeletons of 6-day-old control (left) and *Twist1^flox/+^*; *Twist2^Cre/+^* (right) mice stained with alcian blue (cartilage) and alizarin red (bone). The skeleton of the *Twist1^flox/+^*; *Twist2^Cre/+^* mouse is remarkably smaller. (B) Alcian blue- and alizarin red-stained skull from 6-day-old *Twist1^flox/+^*; *Twist2^Cre/+^* mice (right) showed delayed fusion of interfrontal suture and open posterior fontanel (arrows), compared with the control mice (left). (C) Alcian blue- and alizarin red-stained hind foot of 6-day-old control (left) and *Twist1^flox/+^*; *Twist2^Cre/+^* (right) mice. Note the delayed ossification in metatarsals (mt) and phalanges (pl), and an additional toe (arrow) originating from the same (or duplicated) metatarsal as the hallux in *Twist1^flox/+^*; *Twist2^Cre/+^* mice. (D) Plain X-radiography of the tibiae from 6-day-old control (left) and *Twist1^flox/+^*; *Twist2^Cre/+^* mice (right). The *Twist1^flox/+^*; *Twist2^Cre/+^* mice had shorter tibiae and reduced radiopacity, compared to the control mice. (E) Representative three-dimensional μ-CT images of tibiae from 6-day-old control (left) and *Twist1^flox/+^*; *Twist2^Cre/+^* (right) mice. The *Twist1^flox/+^*; *Twist2^Cre/+^* mice showed reduced trabecular (arrowheads) and cortical bones (arrows). (F–H) Quantitative μ-CT data showing that the 6-day-old *Twist1^flox/+^*; *Twist2^Cre/+^* mice had a significant decrease in the ratio of bone volume (BV)/total volume (TV) (F) and in apparent bone density (G), compared to the control mice (n = 6, P<0.001). The *Twist1^flox/+^*; *Twist2^Cre/+^* mice also presented reduced material density although no statistically significant difference was observed (H).

We first examined the overall skeletal structures of *Twist1^flox/+^*; *Twist2^Cre^* ^/+^ mice in Figure 1. The alcian blue/alizarin red staining showed that *Twist1^flox/+^*; *Twist2^Cre^* ^/+^ mice had a much smaller skeleton with delayed fusion of the interfrontal suture, open posterior fontanelles and delayed ossification in the metatarsals and phalanges (Figs. 1A–C). In addition, the *Twist1^flox/+^*; *Twist2^Cre^* ^/+^ mice developed an extra toe close to the hallux (Fig. 1C), the hallmark of *Twist1* heterozygous mice [32]. Plain X-radiography showed that the *Twist1^flox/+^*; *Twist2^Cre^* ^/+^ mice had reduced radiopacity in the tibiae compared to the control mice (Fig. 1D). Micro-CT images further confirmed that the *Twist1^flox/+^*; *Twist2^Cre^* ^/+^ mice had reduced trabecular bone and decreased cortical bone thickness (Fig. 1E). The quantitative analyses revealed a significant decrease in trabecular bone volume versus total volume (BV/TV) and in apparent bone density in the *Twist1^flox/+^*; *Twist2^Cre^* ^/+^ mice (Figs. 1F–G). The material density was also slightly reduced in the *Twist1^flox/+^*; *Twist2^Cre^* ^/+^ mice although the difference in this parameter was not statistically significant compared to the control mice (Fig. 1H). These data demonstrated that bone formation was inhibited in the *Twist1^flox/+^*; *Twist2^Cre^* ^/+^ mice.

### Defects in osteoblast differentiation in Twist1^flox/+^; Twist2^Cre/+^ mice

**Figure 2:**
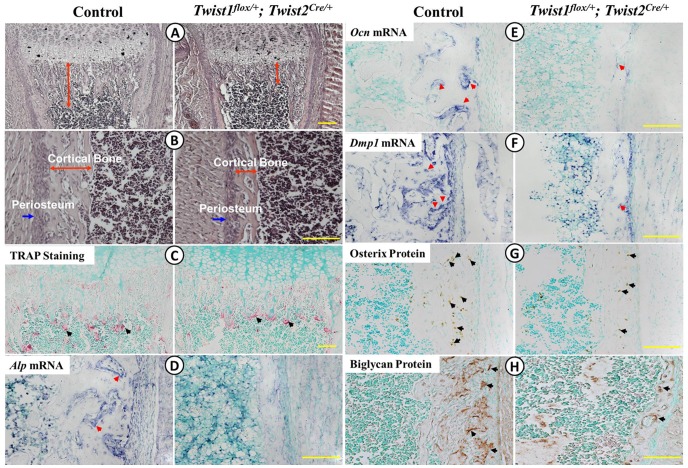
Histological examination of *Twist1^flox/+^*; *Twist2^Cre/+^* mice. (A–B) Femur sections of 6-day-old control and *Twist1^flox/+^*; *Twist2^Cre/+^* mice were stained with H&E. The *Twist1^flox/+^*; *Twist2^Cre/+^* mice displayed reduced metaphyseal trabecular bone (A, red arrows) and a decreased thickness of the periosteum (B, blue arrows) and cortical bone (B, red arrows). (C) TRAP staining of femur sections of 6-day-old control and *Twist1^flox/+^*; *Twist2^Cre/+^* mice. Note that the osteoclasts (red arrows) appeared to be similar in size and distribution in the control and *Twist1^flox/+^*; *Twist2^Cre/+^* mice. The osteoclast densities were 0.55±0.06/0.01 mm^2^ in the controls (*n* = 5) and 0.60±0.02/0.01 mm^2^ in the *Twist1^flox/+^*; *Twist2^Cre/+^* mice (*n* = 5, *P*>0.05). (D–F) *In situ* hybridization analyses (signal in blue) of the transcripts of *Alp* (D), *Ocn* (E) and *Dmp1* (F) in the femurs of one-week-old control and *Twist1^flox/+^*; *Twist2^Cre/+^* mice. (G, H) Immunohistochemical analyses (signal in brown) of the osterix (G) and biglycan (H) protein levels in the femurs of the 6-day-old control and *Twist1^flox/+^*; *Twist2^Cre/+^* mice. Scale bar = 100 µm.

**Figure 3:**
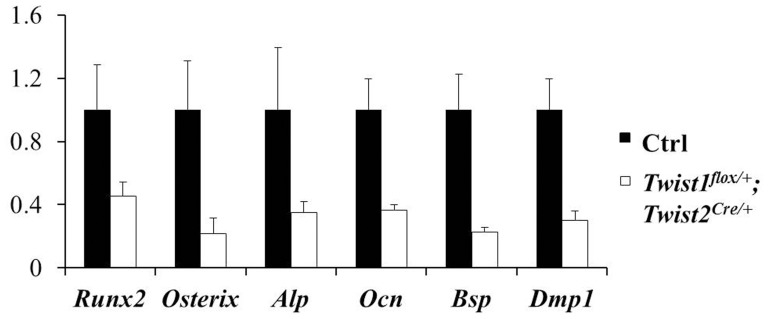
Quantitative real-time PCR analyses of osteoblast differentiation markers. Real-time PCR was performed with total RNA isolated from the long bones of the one-week-old control and *Twist1^flox/+^*; *Twist2^Cre/+^* mice. The expressions of key transcription factors associated with osteoblast differentiation (Runx2 and osterix), osteoblast markers (*Alp*, *Ocn* and *Bsp*) and osteocyte marker (*Dmp1*) were reduced in *Twist1^flox/+^*; *Twist2^Cre/+^* mice. The mRNA levels in the control mice were set as one, and the mRNA levels of *Twist1^flox/+^*; *Twist2^Cre/+^* mice were expressed as folds of those in the control mice. The data represented three analyses (*n* = 3) for each group.

We carried out a series of histological and molecular analyses to determine whether there were any abnormalities in osteoclast numbers and/or osteoblast differentiation. First, H&E staining confirmed that the *Twist1^flox/+^*; *Twist2^Cre^* ^/+^ mice formed less trabecular bone and thinner periosteum and cortical bone (Figs. 2A–B). Second, TRAP staining showed that the distribution and size of the osteoclasts in the *Twist1^flox/+^*; *Twist2^Cre^* ^/+^ mice were similar to those of the control mice (Fig. 2C). Although the osteoclast density was slightly increased in the *Twist1^flox/+^*; *Twist2^Cre^* ^/+^ mice, no significant difference was observed between two groups. Third, we examined the expression levels of the osteoblast differentiation markers by *in situ* hybridization, immunohistochemistry and real-time PCR. These methods revealed that the levels of the osteoblast differentiation markers Alp (Figs. 2D and 3), Ocn (Figs. 2E and 3), biglycan (Fig. 2H) and Bsp (Fig. 3) were sharply reduced in the *Twist1^flox/+^*; *Twist2^Cre^* ^/+^ mice compared to the control mice. In addition, the osteocyte marker Dmp1 was also dramatically decreased (Fig. 2F and 3). Furthermore, the expression levels of Runx2 and Osx, two key transcription factors essential for osteoblast differentiation and bone formation, were remarkably reduced in the *Twist1^flox/+^*; *Twist2^Cre^* ^/+^ mice, compared to the control mice (Figs. 2G and 3). Taken together, these findings supported the hypothesis that the reduced bone formation resulted from defects in osteoblast differentiation rather than abnormal osteoclast activities.

### Reduced cell proliferation in Twist1^flox/+^; Twist2^Cre/+^ mice

**Figure 4:**
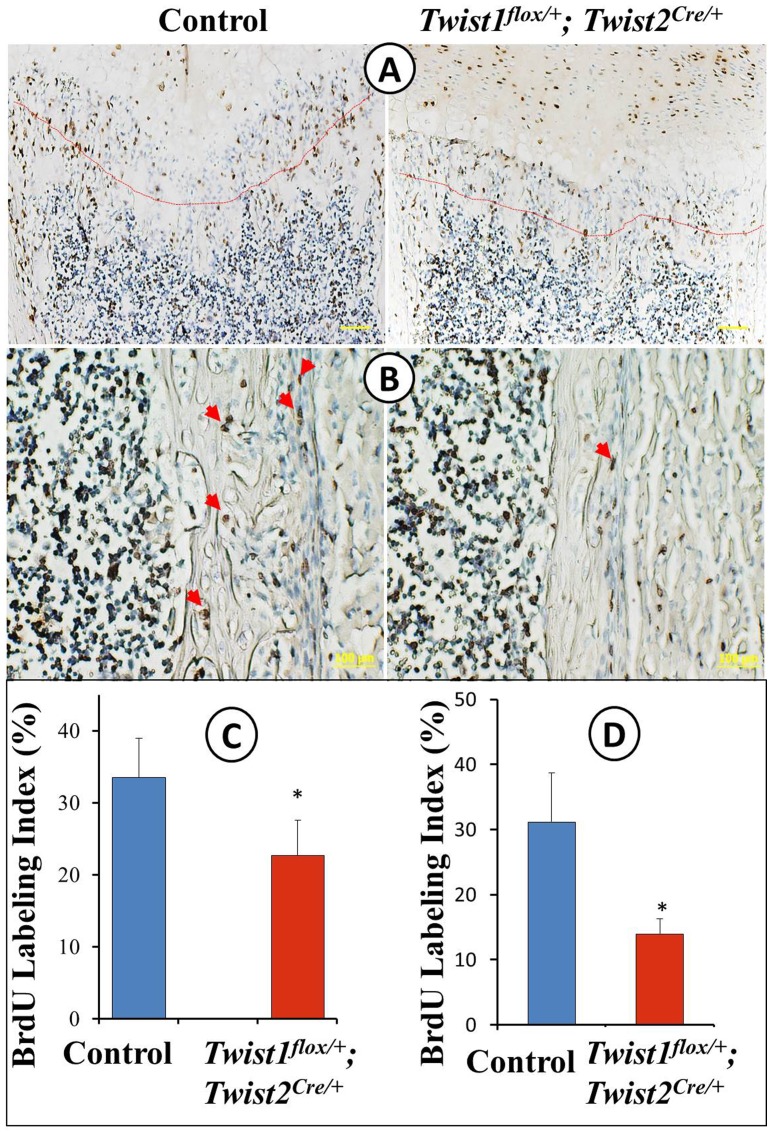
Cell proliferation was reduced in the *Twist1^flox/+^*; *Twist2^Cre/+^* mice. (A–B) BrdU immunohistochemical staining of the femur sections of 7-day-old control and *Twist1^flox/+^*; *Twist2^Cre/+^* mice. The BrdU-positive cells (signal in brown) were counted in a 100-µm zone of the metaphysis, demarcated by the chondro-osseous junction and the marked line (A), and in the femoral diaphysis (B). The osteoblast/osteoprogenitor proliferation was reduced in both the metaphysis (C) and diaphysis (D) in the *Twist1^flox/+^*; *Twist2^Cre/+^* mice, compared to the control mice (*n* = 4, *P<0.05). The data were expressed as the percentage of BrdU-positive nuclei versus total nuclei. Scale bars: 100 µm.

Since the *Twist1^flox/+^*; *Twist2^Cre^* ^/+^ mice had a reduced periosteal layer compared to the control mice (Fig. 2B), we performed BrdU labeling to determine whether the proliferation of osteoblasts and their progenitors was affected. We noticed that the BrdU-positive cells in the area of the metaphysis (Figs. 4A), as well as in the mid diaphyseal periosteum and cortical bone (Fig. 4D), were significantly reduced in the *Twist1^flox/+^*; *Twist2^Cre^* ^/+^ mice. However, no difference in the osteoblast apoptosis was observed (Figure S2). These findings suggested that the reduced cell proliferation might also contribute to the reduced bone formation in the *Twist1^flox/+^*; *Twist2^Cre^* ^/+^ mice.

### Reduced FGF signaling in Twist1^flox/+^; Twist2^Cre/+^ mice

**Figure 5:**
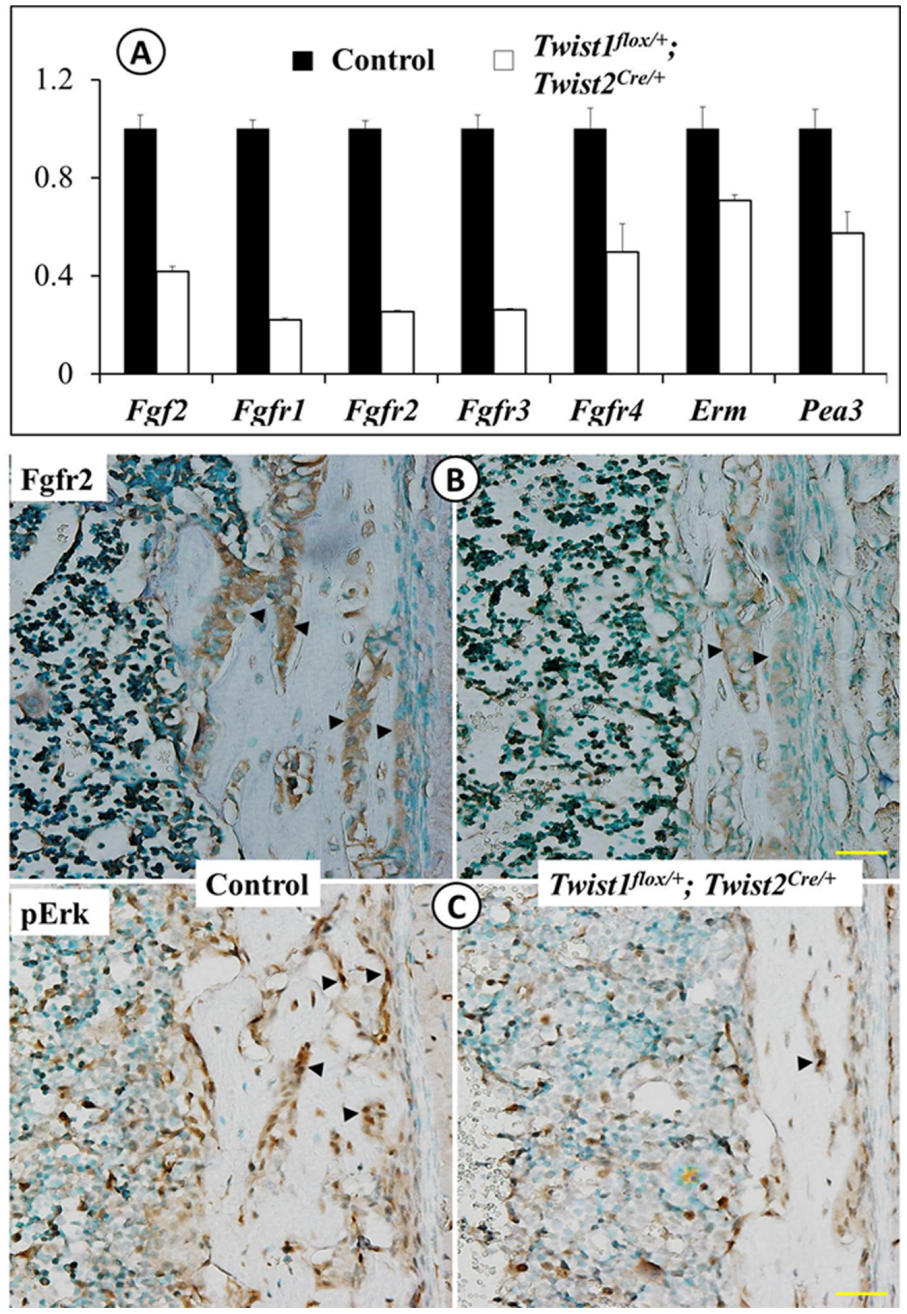
Reduced FGF signaling in *Twist1^flox/+^*; *Twist2^Cre/+^* mice. (A) Quantitative real-time PCR was performed to analyze the mRNA levels of *Fgf2*, *Fgfr1*, *Fgfr2*, *Fgfr3* and *Fgfr4*, as well as two effector molecules, *Erm* and *Pea3*, using total RNA isolated from the long bones of 8-day-old control and *Twist1^flox/+^*; *Twist2^Cre/+^* mice. The mRNA levels in the control mice were set as one, and the mRNA levels of *Twist1^flox/+^*; *Twist2^Cre/+^* mice were expressed as folds of those in the control mice. The data represented three analyses (*n* = 3) for each group. (B) Immunohistochemistry showed that the level of the Fgfr2 protein (signal in brown) was reduced in the femurs of the *Twist1^flox/+^*; *Twist2^Cre/+^* mice (right), compared to the control mice (left). (C) Immunohistochemistry showed that there was less phospho-Erk1/2 (signal in brown) than in the femurs of *Twist1^flox/+^*; *Twist2^Cre/+^* mice, compared to the control mice. Scale bars: 100 µm.

A variety of studies have indicated interactions between Twist1 and FGF signaling although the outcome of such interactions appears to be context dependent [15], [17], [18], [33]. We analyzed the components of the FGF signaling pathway in the *Twist1^flox/+^*; *Twist2^Cre^* ^/+^ mice. Quantitative real-time PCR revealed a substantial decrease in the transcript levels of *Fgf2* and *Fgfrs1*, *2*, *3* and *4* in the *Twist1^flox/+^*; *Twist2^Cre^* ^/+^ mice (Fig. 5A). Immunohistochemistry further confirmed that the Fgfr2 protein was markedly reduced in the long bones of the *Twist1^flox/+^*; *Twist2^Cre^* ^/+^ mice (Fig. 5B). FGF signaling triggered the MAP kinase cascade, resulting in the phosphorylation and activation of p44/42 (Erk1/2) and the expression of two effector molecules, Erm and Pea3 [34]. Consistent with the reduced *Fgf2* and *Fgfr* expression, the immunohistochemistry revealed that the levels of phospho-Erk1/2 were considerably lower in *Twist1^flox/+^*; *Twist2^Cre^* ^/+^ mice than in the control mice (Fig. 5C). Accordingly, quantitative real-time PCR demonstrated that the levels of *Erm* and *Pea3* transcripts were significantly downregulated (Fig. 5A). These data demonstrated that FGF signaling was reduced in the *Twist1^flox/+^*; *Twist2^Cre^* ^/+^ mice, suggesting that Twist1 and Twist2 might upregulate the expressions of *Fgf2* and *Fgfrs*.

### Twist1 and Twist2 stimulated Fgfr2 promoter activity

**Figure 6:**
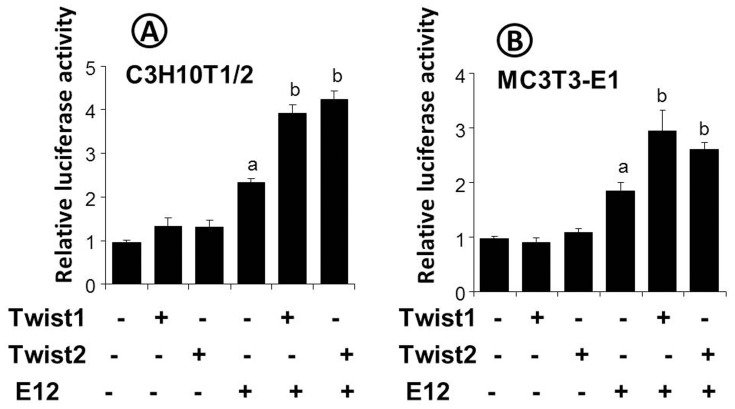
Effects of Twist1, Twist2 and E12 on the activity of a 4.9*Fgfr2* promoter fragment. C3H10T1/2 (A) and MC3T3-E1 cells (B) were transiently co-transfected with a 4.9 kb *Fgfr2* promoter luciferase construct and the indicated expression constructs, along with a pRL-TK construct as an internal control. The luciferase activities were determined by a dual luciferase assay system, and the promoter activities were expressed as luciferase activities relative to that of the control. The values represented mean ± SD. n = 3 for each group. “a” indicates significant difference from the control (p<0.05); “b” denotes a significant difference from all other groups (p<0.05).

As described above, Fgfr2 was significantly downregulated at both the mRNA and protein levels in the *Twist1^flox/+^*; *Twist2^Cre^* ^/+^ mice. The skeletal phenotype of *Twist1^flox/+^*; *Twist2^Cre^* ^/+^ mice partially resembles that of the *Fgfr2* conditional knock-out mice [22]. Therefore, we examined whether Twist1 and Twist2 could upregulate the *Fgfr2* promoter activity *in vitro*. Our promoter luciferase reporter assay revealed that Twist1 or Twist2 alone was unable to significantly stimulate a 4.9 kb *Fgfr2* promoter fragment. However, they strongly enhanced the stimulatory activity of E12, a ubiquitously expressed Twist binding partner, in both the C3H10T1/2 mesenchymal cells and MC3T3-E1 pre-osteoblast cells (Fig. 6). This *in vitro* evidence implied that Twist1 and Twist2, together with E12, could regulate the *Fgfr2* expression.

## Discussion

Twist1 and Twist2 are two highly conserved members of the Twist subfamily of bHLH transcription factors. In this study, we generated *Twist1-* and *Twist2-*haploinsufficient mice (*Twist1^flox/+^*; *Twist2^Cre^* ^/+^), and found that these mice displayed delayed closure of the cranial sutures. Furthermore, we found that the *Twist1^flox/+^*; *Twist2^Cre^* ^/+^ mice presented with reduced bone formation and impaired osteoprogenitor proliferation and osteoblast differentiation as well as compromised FGF signaling.

Previous studies have demonstrated that the cranial sutures of the Twist1 heterozygous mice prematurely fuse [10], [11], [32]. To confirm this finding, we crossed the *Twist1-*floxed mice (*Twist1^flox/flox^*) with *Sox2-Cre* transgenic mice expressing a Cre recombinase ubiquitously in the epiblast cells at embryonic day 6.5 and generated conditional *Twist1-*haploinsufficient mice (*Twist1^flox/+^*; *Sox2-Cre*) (Figure S3). We found that the *Twist1^flox/+^*; *Sox2-Cre* mice developed craniosynostosis - a phenotype that is similar to the *Twist1* heterozygous mice [11], [32], characterized by much narrower sagittal and interfrontal sutures and had additional toe originating from a duplicated metatarsal as demonstrated by alcian blue and alizarin red staining (Figure S3A–D). The plain X-radiography and histological examination did not reveal apparent difference in the radiopacity of the long bones, in the metaphyseal trabecular bones or in the diaphyseal cortical bones between *Twist1^flox/+^*; *Sox2-Cre* and control mice (Figure S3E–G). In contrast, although the *Twist2* heterozygous mice are viable and fertile with no apparent phenotype [20], [31], the *Twist1^flox/+^*; *Twist2^Cre^* ^/+^ mice showed reduced growth of both cranial and long bones as well as delayed closure of the cranial sutures. Therefore, the *Twist1^flox/+^*; *Twist2^Cre^* ^/+^ mice displayed a skeletal phenotype that is opposite to that of *Twist1* heterozygous mice.

Our current studies and the studies from other labs have demonstrated that Twist1 and Twist2 share similar *in vitro* and *in vivo* functions [11]. Therefore, the phenotypic difference between single *Twist1/2*-heterozygous mice and *Twist1^flox/+^*; *Twist2^Cre^* ^/+^ mice might be due to the overlapping expression patterns of *Twist1* and *Twist2* during mouse embryonic development [6]. Both *Twist1* and *Twist2* are highly expressed in the condensed mesenchyme that later gives rise to osteoblasts and chondrocytes [6], [11], [22]. Thus, the outcomes of the loss of Twist1 and/or Twist2 really depend on the relative expression of both genes in a specific tissue.

Accumulating evidence supports the notion that Twist1 might control cranial suture development through modulating FGF signaling. It was found that the mutations in FGF receptors FGFR1, FGFR2, and FGFR3 in humans are associated with craniosynostosis, a characteristic phenotype of the Saethre-Chotzen Syndrome caused by dominant loss-of-function TWIST1 mutations [35]. In addition, the primary cranial osteoblasts isolated from SCS patients with Twist1 mutations show reduced *FGFR2* transcript levels, which can be restored by overexpression of TWIST1 [18]. Twist1 haploinsufficiency in mice also results in an altered *Fgfr2* expression pattern in the cranial sutures [17]. It has been proposed that Twist1 haploinsufficiency favors the formation of Twist1 homodimers in the osteogenic front of cranial sutures, which results in the upregulation of *Fgfr2* expression and leads to craniosynostosis [16]. Taken together, the data from these human and mouse studies suggest that Twist1 might regulate FGF signaling, particularly the *Fgfr2* expression, in a context-dependent manner.

Our current studies provide further support that Twist1 and Twist2 regulate FGF signaling in bone formation. We found that *Twist1^flox/+^*; *Twist2^Cre^* ^/+^ mice had reduced FGF signaling in bone as a result of decreased expression of not only *Fgfr2* but also *Fgfr1*, *Fgfr3*, *Fgfr4* and even *Fgf2*. FGF signaling plays essential roles throughout osteogenesis, including the commitment of mesenchymal cells to osteoprogenitors, the proliferation and differentiation of osteoprogenitors to osteoblasts, and osteoblast apoptosis [22], [36]. Indeed, the *Twist1^flox/+^*; *Twist2^Cre^* ^/+^ mice displayed reduced proliferation of osteoprogenitor cells and defective osteoblast differentiation. Some of the skeletal abnormalities even resemble those of *Fgfr2* conditional knockout mice [22]. Consistent with the *in vivo* results, our *in vitro* promoter luciferase assays further supported the role of Twist1 and Twist2 in the upregulation of *Fgfr2* promoter activity when E12 was present. However, it remains to be determined why only Twist/E12 heterodimers, instead of Twist homodimers, stimulate *Fgfr2* promoter activity.

In summary, our current study suggested that Twist1 and Twist2 may synergistically enhance the proliferation and differentiation of osteoprogenitors via the upregulation of FGF signaling during skeletal development. However, further studies are necessary to determine whether these two genes perform identical functions and could completely replace each other *in vivo*. Research is also needed to advance our understanding of how the expression levels of Twist1 and Twist2 are temporally and spatially regulated during development and how fine-tuning achieves the optimal Twist protein level needed for normal skeletal development. Such knowledge is essential for the development of future therapies aimed at correcting the effects of Twist deficiency in humans.

## Supporting Information

Figure S1 **The mRNA levels of** ***Twist1*** **and** ***Twist2*** **in the long bones of** ***Twist1^flox/+^*** **;** ***Twist2^Cre/+^*** **mice.** Real-time PCR was performed with total RNA isolated from the long bones of the 8-day-old control and *Twist1^flox/+^*; *Twist2^Cre/+^* mice. The primers used for *Twist1* were sense 5′- CAGCGGGTCATGGCTAAC-3′ and antisense 5′- GCAGGACCTGGTACAGGAAG-3′, and for *Twist2* sense 5′- AGCAAGAAATCGAGCGAAGA-3′ and antisense 5′- CAGCTTGAGCGTCTGGATCT-3′. The mRNA levels of *Twist1* and *Twist2* were about three folds less in the *Twist1^flox/+^*; *Twist2^Cre/+^* mice than in the control mice. The data represented three analyses (*n* = 3) for each group.
(TIF)

Figure S2 **Osteoblast apoptosis in the** ***Twist1^flox/+^*** **;** ***Twist2^Cre/+^*** **mice.** TUNEL assay was used to analyze the osteoblast apoptosis in the long bones of 6-day-old *Twist1^flox/+^*; *Twist2^Cre/+^* mice and control mice. Three serial sections from each of four individual *Twist1^flox/+^*; *Twist2^Cre/+^* mice and control littermates were counted. No significant difference in osteoblast apoptosis was found between the two groups of mice.
(TIF)

Figure S3 **Skeletal abnormalities of** ***Twist1^flox/+^*** **;** ***Sox2-Cre*** **mice.** (A) The skeletons of 7-day-old *Twist1^flox/+^* (control) and *Twist1^flox/+^*; *Sox2-Cre* mice were stained with alcian blue and alizarin red. (B–C) Alcian blue and alizarin red stained skulls, femurs and tibiae, and hind feet from 7-day-old control mice and *Twist1^flox/+^*; *Sox2-Cre* mice; The *Twist1^flox/+^*; *Sox2-Cre* mice showed much narrower sagittal and interfrontal sutures (arrows; B) and had additional toe (arrow) originating from a duplicated metatarsal (MT; D), but the femurs and tibiae showed no apparent difference between two groups (C). (E) Plain X-radiography of the hind limbs from 7-day-old control and *Twist1^flox/+^*; *Sox2-Cre* mice. No apparent difference was noted between the two groups of mice. (F and G) Histological examination of *Twist1^flox/+^*; *Sox2-Cre* mice. Tibia sections of 7-day-old control and *Twist1^flox/+^*; *Sox2-Cre* mice were stained with H&E. No apparent difference was observed in the metaphyseal trabecular bone (F) or in the diaphyseal cortical bone (G) between these two groups.
(TIF)

Materials and Methods S1 **Generation of** ***Twist1^flox/+^*** **;** ***Sox2-Cre*** **mice.**
(DOCX)
