# Supplementary figures and images for: Twist1- and Twist2-Haploinsufficiency Results in Reduced Bone Formation
Source: PLoS One. 2014 Jun 27;9(6):e99331. doi: 10.1371/journal.pone.0099331 (PMC4074031; doi:10.1371/journal.pone.0099331)

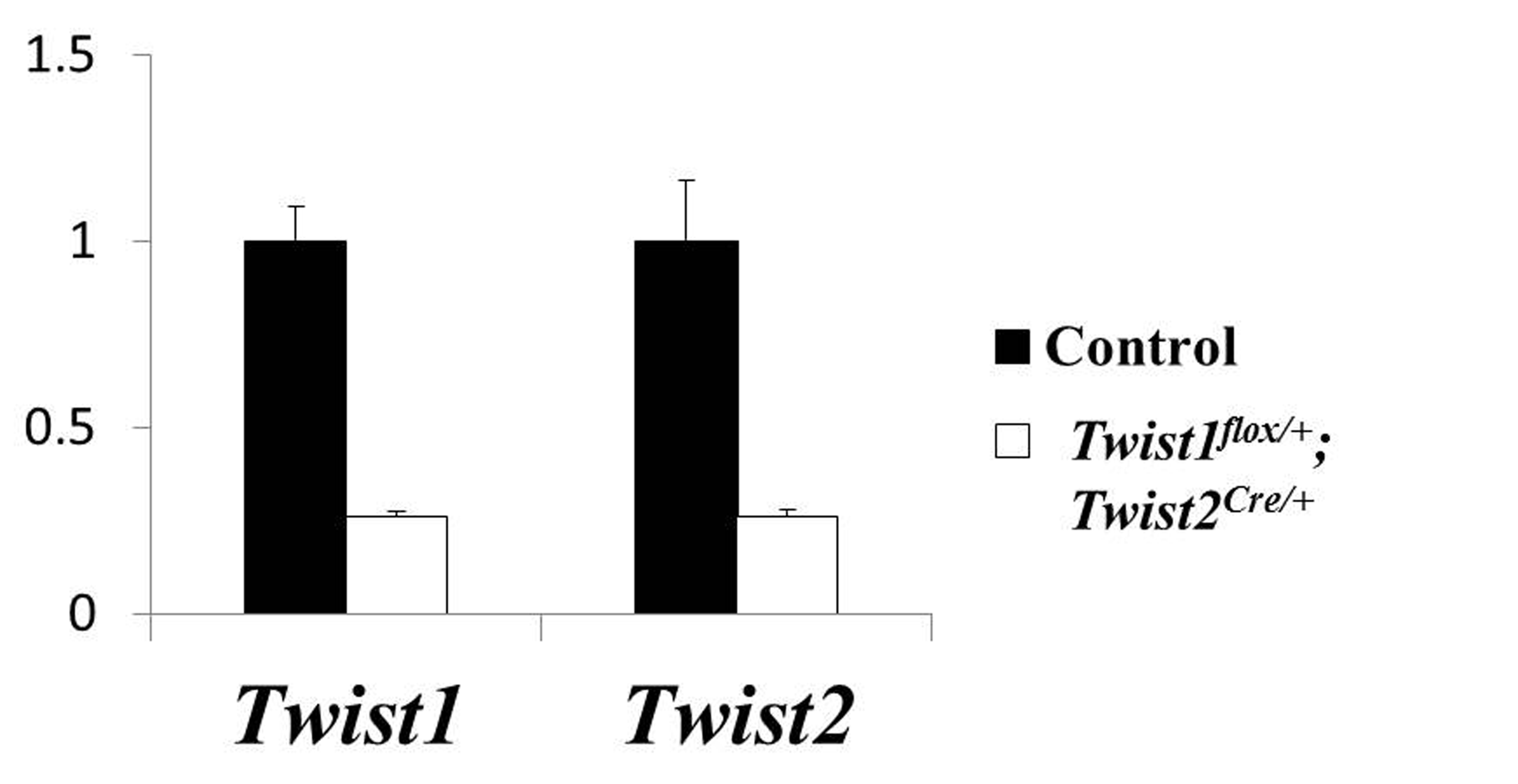

Supplement: Figure S1 — The mRNA levels of Twist1 and Twist2 in the long bones of Twist1flox/+ ; Twist2Cre/+ mice. Real-time PCR was performed with total RNA isolated from the long bones of the 8-day-old control and Twist1flox/+; Twist2Cre/+ mice. The primers used for Twist1 were sense 5′- CAGCGGGTCATGGCTAAC-3′ and antisense 5′- GCAGGACCTGGTACAGGAAG-3′, and for Twist2 sense 5′- AGCAAGAAATCGAGCGAAGA-3′ and antisense 5′- CAGCTTGAGCGTCTGGATCT-3′. The mRNA levels of Twist1 and Twist2 were about three folds less in the Twist1flox/+; Twist2Cre/+ mice than in the control mice. The data represented three analyses (n = 3) for each group. (TIF) [file pone.0099331.s001.tif]

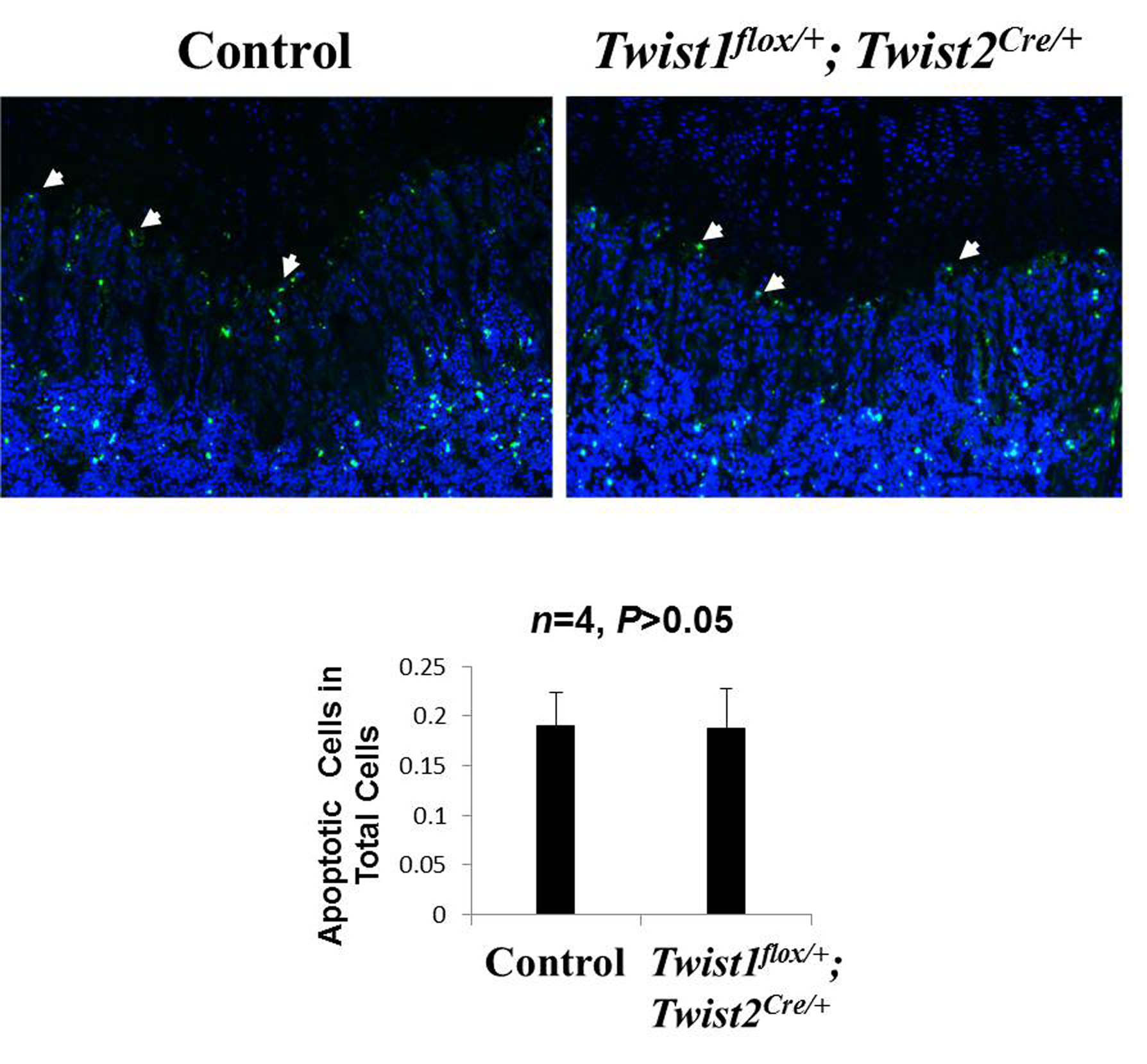

Supplement: Figure S2 — Osteoblast apoptosis in the Twist1flox/+ ; Twist2Cre/+ mice. TUNEL assay was used to analyze the osteoblast apoptosis in the long bones of 6-day-old Twist1flox/+; Twist2Cre/+ mice and control mice. Three serial sections from each of four individual Twist1flox/+; Twist2Cre/+ mice and control littermates were counted. No significant difference in osteoblast apoptosis was found between the two groups of mice. (TIF) [file pone.0099331.s002.tif]

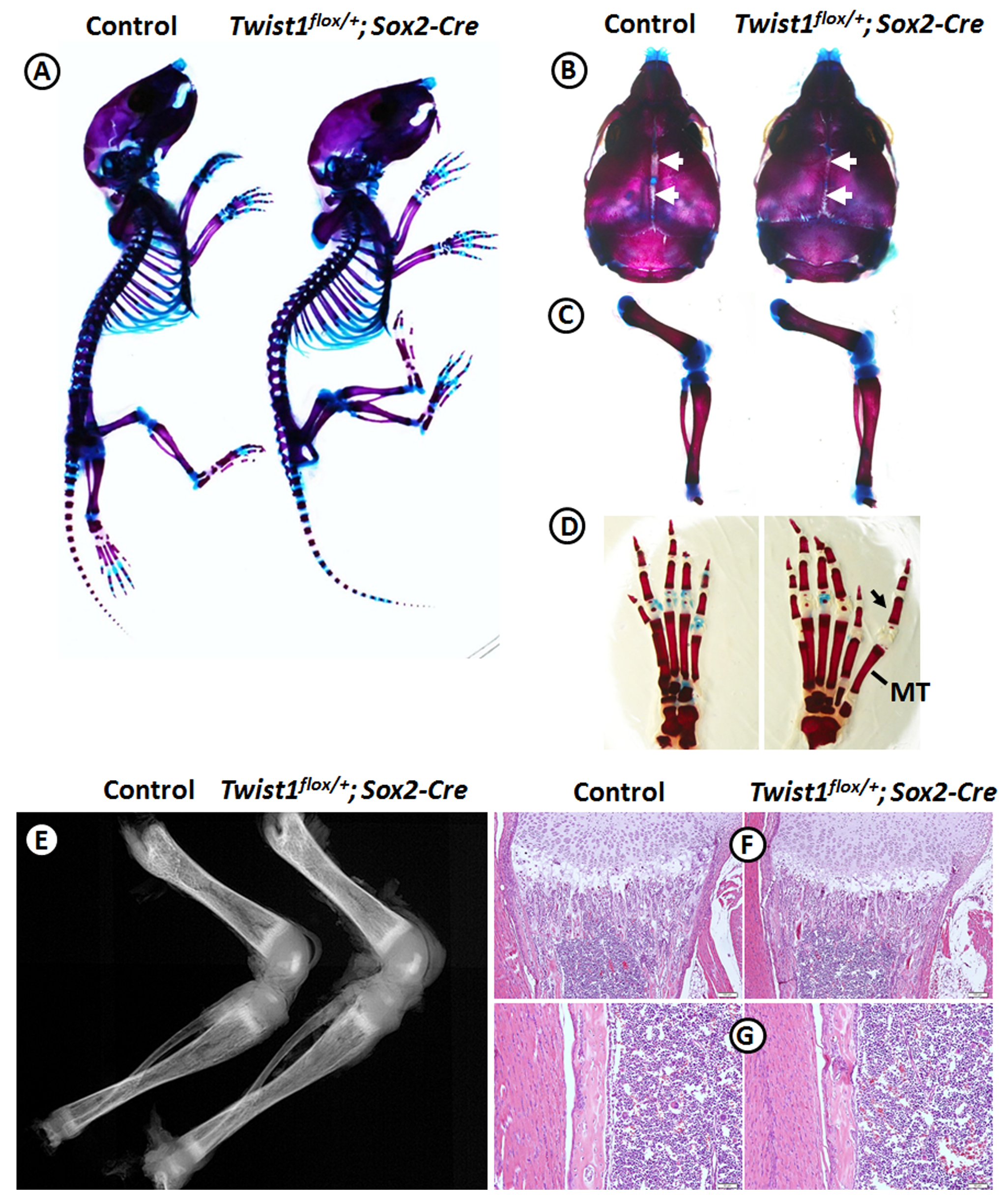

Supplement: Figure S3 — Skeletal abnormalities of Twist1flox/+ ; Sox2-Cre mice. (A) The skeletons of 7-day-old Twist1flox/+ (control) and Twist1flox/+; Sox2-Cre mice were stained with alcian blue and alizarin red. (B–C) Alcian blue and alizarin red stained skulls, femurs and tibiae, and hind feet from 7-day-old control mice and Twist1flox/+; Sox2-Cre mice; The Twist1flox/+; Sox2-Cre mice showed much narrower sagittal and interfrontal sutures (arrows; B) and had additional toe (arrow) originating from a duplicated metatarsal (MT; D), but the femurs and tibiae showed no apparent difference between two groups (C). (E) Plain X-radiography of the hind limbs from 7-day-old control and Twist1flox/+; Sox2-Cre mice. No apparent difference was noted between the two groups of mice. (F and G) Histological examination of Twist1flox/+; Sox2-Cre mice. Tibia sections of 7-day-old control and Twist1flox/+; Sox2-Cre mice were stained with H&E. No apparent difference was observed in the metaphyseal trabecular bone (F) or in the diaphyseal cortical bone (G) between these two groups. (TIF) [file pone.0099331.s003.tif]
