# Supplementary material for: Twist1- and Twist2-Haploinsufficiency Results in Reduced Bone Formation
Source: PLoS One. 2014 Jun 27;9(6):e99331. doi: 10.1371/journal.pone.0099331 (PMC4074031; doi:10.1371/journal.pone.0099331)
Supplement: Materials and Methods S1 — Generation of Twist1flox/+ ; Sox2-Cre mice. (DOCX) [file pone.0099331.s004.docx]

**Materials and Methods**

**Generation of *Twist1^flox/+^*; *Sox2-Cre* mice**

*Twist1^flox/flox^* mice were mated with *Sox2-Cre* transgenic mice to generate *Twist1*-haploinsufficient mice *Twist1^flox/+^*; *Sox2-Cre* mice. The *Sox2-Cre* mice were maintained on a C57BL/6N genetic background and obtained from the Jackson Laboratory. These transgenic mice bear a cre recombinase gene under the control of a Sox2 promoter, and express a Cre recombinase ubiquitously in the epiblast cells at embryonic day 6.5. Therefore, the floxed *Twist1* allele is deleted in all the body tissues of the *Twist1^flox/+^*; *Sox2-Cre* mice. The skeletal phenotype of 7-day-old *Twist1^flox/+^*; *Sox2-Cre*  mice were analyzed by alcian blue/alizarin red staining, plain X-ray radiography and Hematoxylin and Eosin (H&E) staining as described in the main manuscript. The age-matched *Twist1^flox/+^* mice were used as controls.
